# Randomised controlled trial of neurostimulation for symptoms of anorexia nervosa (TRENA study): study protocol

**DOI:** 10.1186/s40337-023-00940-7

**Published:** 2023-12-08

**Authors:** Anna J. Harvey, Sloane Madden, Anthony Rodgers, Michael Bull, Mary Lou Chatterton, Dusan Hadzi-Pavlovic, Colleen K. Loo, Donel M. Martin

**Affiliations:** 1 University of New South Wales, Sydney, NSW Australia; 2 Black Dog Institute, Sydney, NSW Australia; 3 Ramsay Clinic Northside, Sydney, NSW Australia; 4 The George Institute for Global Health, Sydney, NSW Australia; 5 Monash University, Melbourne, VIC Australia

**Keywords:** Anorexia nervosa, Eating disorder, Transcranial direct current stimulation, Repetitive transcranial magnetic stimulation

## Abstract

**Background:**

Anorexia nervosa (AN) has amongst the highest mortality rates and the highest treatment costs of any psychiatric disorder. Recently, interest in non-invasive brain stimulation as a novel treatment for AN has grown. These include repetitive transcranial magnetic stimulation (rTMS) and transcranial direct current stimulation (tDCS).

**Methods:**

This double-blind, randomised sham-controlled trial will compare the relative acceptability and efficacy of tDCS and rTMS in people with AN. 70 participants will be randomised to active or sham tDCS, or active or sham rTMS treatment (2:1:2:1 ratio) over an 8-week treatment period. Participants will receive treatment as usual across the study duration. The primary outcomes are change on the Eating Disorder Examination Questionnaire and treatment acceptability. Secondary outcomes will include change in weight, cognition, mood, interpersonal functioning, and quality of life. Following the 8-week assessment, all participants will have the option of receiving an additional 12 weeks of at-home tDCS. A follow-up assessment will be conducted at 20 weeks post treatment.

**Discussion:**

Research into non-invasive brain stimulation as treatments for AN has potential to improve clinical outcomes for patients by comparing the relative efficacy and acceptability of both treatment modalities in the inpatient and at-home setting (i.e., for at-home tDCS) results from this study will provide important information for informing future larger clinical trials of these treatments for AN.

***Trial registration*:**

ClinicalTrials.gov Identifier: NCT05788042.

## Background

Anorexia nervosa (AN) is a life-threatening eating disorder characterised by a restriction of energy intake relative to needs leading to a significantly low weight [4]. Lifetime prevalence rates are up to 4% among females and 0.3% among males [73], with age of onset predominantly in adolescence [48]. AN has amongst the highest mortality rate of any psychiatric disorder [5]. Complications from malnutrition affect all body systems [31] including growth retardation, osteoporosis, infertility and changes in brain structure [43]. AN is also associated with psychological complications including mood disorders, anxiety disorders, obsessive compulsive disorder, substance abuse, and personality disorders [37, 56]. Moreover, individuals with AN exhibit cognitive impairment, with severity correlating with degree of malnutrition and chronicity [34]. Although, specialised family-based psychology treatments are beneficial for adolescents [17, 32, 51]; psychotherapy and pharmacotherapy interventions for adults with AN, have limited efficacy with reports of 70–90% of patients not achieving full remission [12, 24, 25, 39, 68, 69, 77] with no significant advantages of combining these treatments [6, 10, 74]. The investigation of novel treatment strategies is thus critical.

Recently, interest in the therapeutic potential of non-invasive brain stimulation as a novel treatment for AN has grown. Preliminary evidence has suggested that both transcranial direct current stimulation (tDCS) and repetitive transcranial magnetic stimulation (rTMS) targeting the dorsolateral prefrontal cortex (DLPFC) can reduce core eating disorder symptoms in AN [29].

### Transcranial direct current stimulation (tDCS) for people with AN

Transcranial direct current stimulation (tDCS) is a safe, non-invasive brain stimulation technique that involves the passing of a weak direct electrical current (typically 1–2 mA) through the cerebral cortex via electrodes placed upon the scalp. Animal and human studies have shown that tDCS changes the excitability of neurons in stimulated regions in a polarity dependent manner, causing changes in spontaneous neural activity that outlast the period of stimulation [50]. Meta-analyses of randomised controlled trials (RCTs) have demonstrated significant therapeutic benefit in depression [63] and acute cognitive enhancing effects in healthy and clinical samples [8, 26]. tDCS has relative advantages compared to other non-invasive brain stimulation methods, including reduced costs for equipment and the ability for remotely supervised at-home treatment using protocols developed by our team [3]. To date, one double blind RCT [7] has examined the therapeutic potential of tDCS in AN. In this study 43 participants with AN were randomly assigned to receive active or sham tDCS over the left DLPFC over ten 30-min sessions. Although the results did not show major improvements in AN psychopathology or weight recovery compared with sham tDCS, active tDCS reduced the need for excessive control over food intake and improved body image. In addition, three small open-label studies [16, 45, 71] and one case report [67] have examined tDCS efficacy of left DLPFC anodal stimulation in people with AN. Although these studies have varied in terms of treatment protocols (e.g., treatment administered three times a week over 6 weeks or once and twice per day over ten days), all studies suggested benefits for core eating disorder symptoms with the pilot studies showing maintained benefits at 1 month follow up post end acute treatment. Although preliminary, these studies have suggested that anodal tDCS targeting the left DLPFC may be beneficial for improving AN symptoms, though larger RCTs are required.

### Repetitive transcranial magnetic stimulation (rTMS) for people with AN

Repetitive transcranial magnetic stimulation (rTMS) involves the repeated application of a strong, highly localised magnetic field to a small cortical area, depolarising neurons in the stimulated target area and producing downstream neuromodulatory effects [46]. The most studied psychiatric application of rTMS has been for depression that is treatment-resistant, or difficult-to-treat [59]. In 2008, rTMS gained US FDA clearance for treatment-resistant depression. Other psychiatric and neurological disorders approved and treated with rTMS in other jurisdictions include obsessive compulsive disorder, refractory schizophrenia, and Alzheimer's disease. rTMS treatment may also have cognitive enhancing effects. A systematic review and meta-analysis of rTMS cognitive effects in depression found significant cognitive enhancement compared to sham treatment [55].

To date, one double-blind sham-controlled RCT [20, 21] has examined the effects of rTMS in people with severe enduring AN (i.e., illness duration ≥ 3 years). In that study, 34 patients were randomly assigned to receive active or sham rTMS administered to the left DLPFC over 20 consecutive weekdays. Results showed that rTMS was safe and well tolerated. Importantly, clinical benefits with active rTMS were observed, with moderate to large sized improvements for psychological symptoms (anxiety and depression) and medium sized improvement in quality of life at a 3 month follow up. Moreover, an open 18 months follow up showed further improvement in eating disorder symptoms and maintained effects on mood [22]. Several open label studies [23, 28, 47, 58, 76] and case studies [14, 27, 41, 57] have also observed promising therapeutic effects with AN symptoms, with the majority of studies targeting the left DLPFC. Three pilot studies though have explored the efficacy of alternative treatment targets, the dorsomedial prefrontal cortex (DMPFC; Dunlop et al. [28]; Woodside et al. [76]) and the insula [47] with similar promising therapeutic effects for core AN symptoms. Interestingly, a recent secondary analysis of a feasibility study which involved 26 participants with AN who received 20 rTMS treatments targeting the left DLPFC over four weeks found preliminary evidence of greater improvement in core AN symptoms at 3 month follow up in the participants who took concurrent antidepressant medications [23]. In the one trial which examined longer-term outcomes, McClelland et al. [58] found that 3/5 patients had sustained clinical benefits for core AN symptoms (i.e., reduced urge to restrict food intake and the feeling of being full or fat) following a 4-week treatment course at a 12-month follow up. Taken together these studies indicate that that rTMS is safe and well tolerated treatment for AN, however, further research is required to investigate optimal treatment protocols with larger RCTs also required to determine treatment efficacy.

### Current study

Despite initial promising findings, the potential therapeutic benefits of tDCS and rTMS treatment for AN remain unclear. To date, tDCS and rTMS studies have varied in terms of treatment protocols (e.g., administered once or twice per day, up to 20 treatment sessions) and indicated limited efficacy and acceptability of both treatments. Further no study has directly compared the efficacy and acceptability of tDCS and rTMS. Here we propose to conduct the first double-blinded, randomised sham-controlled study to directly compare the therapeutic efficacy and acceptability of these two novel treatment modalities. This study will extend findings from previous studies in several ways, including: (1) using self-administered instead of in-clinic administered tDCS which has potential advantages for people living remotely or with restricted access to health care services; (2) using a newer form of patterned rTMS (intermittent theta burst stimulation: iTBS) which is much quicker to administer than standard rTMS; (3) administering an increased number of treatments and longer sham-controlled treatment period (8 weeks) compared to the only prior RCT of rTMS in AN (20 treatments) and previous studies (up to 4 weeks of acute treatment only); (4) the option of longer-term continuation at-home tDCS treatment to examine the potential for longer-term clinical benefits; (5) exploring participants’ experience of treatment though qualitative interview; and (6) extending the field of treatment strategies for AN expected outcomes including data on the relative efficacy, acceptability and cost effectiveness for both treatment modalities in the inpatient and at-home setting (i.e., for at-home tDCS). This will be important for informing a future larger definitive multicentre clinical trial. This pilot study’s main primary hypotheses are that both active treatment arms will produce clinical improvement in eating disorder psychopathology (measured with Eating Disorder Examination Questionnaire; EDE-Q; Fairburn [30]) at the end of 8-week randomised controlled period and that both active treatments will have high acceptability (measured with number of completed sessions for active tDCS and active rTMS in the acute 8-week RCT period).

## Methods

### Design

**Fig. 1:**
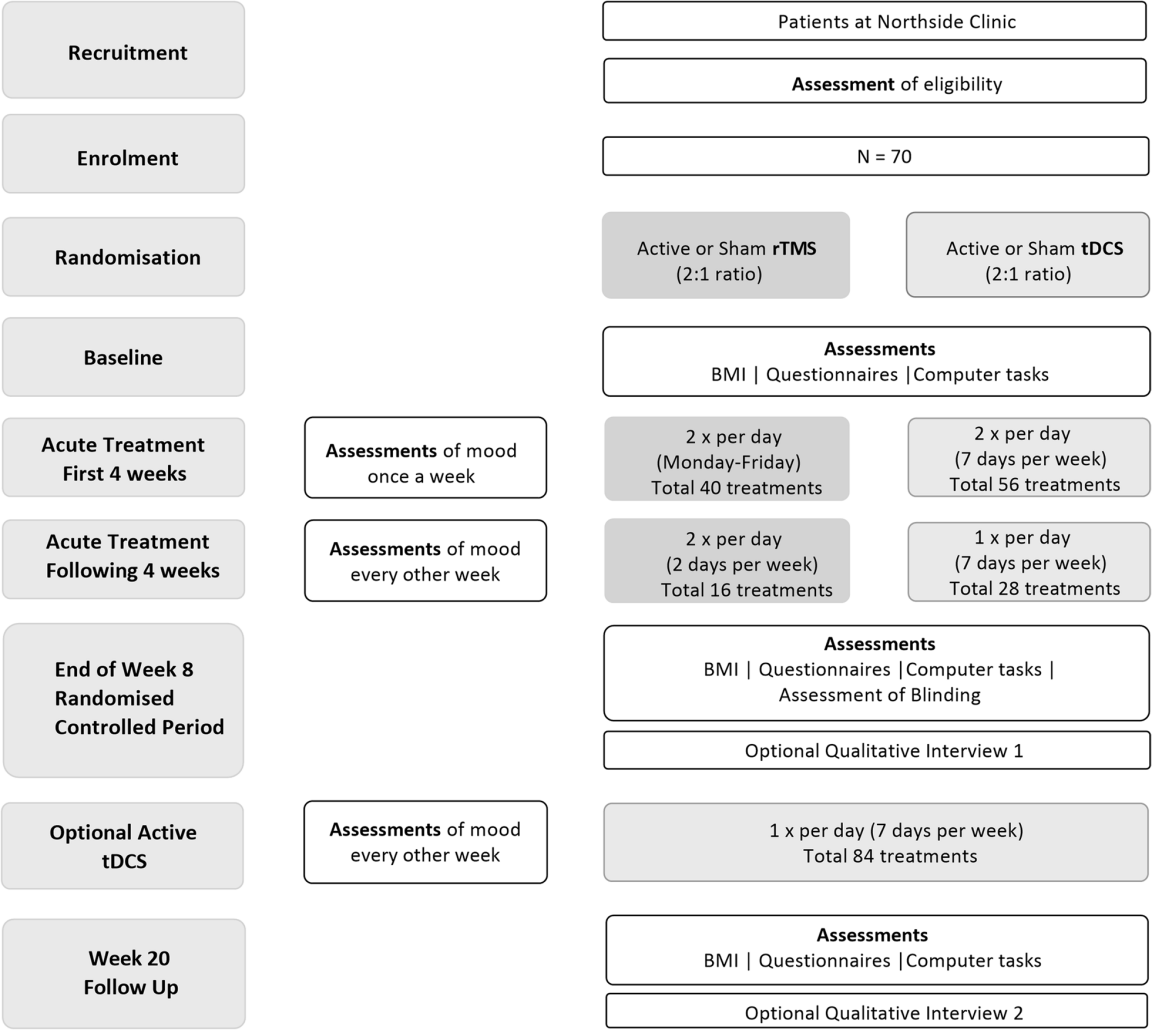
Schematic diagram of TRENA study procedures

This double-blind, randomised, sham-controlled trial will compare rTMS and tDCS acceptability and clinical outcomes in patients with AN. This study protocol was prepared in accordance with the SPIRIT and CONSORT guidelines. Participants will be randomised to one of four groups (2:1:2:1 ratio): active or sham tDCS, or active or sham rTMS. The brain stimulation protocols involve 84 sessions of tDCS (active or sham) or 56 sessions of rTMS (active or sham) over an 8 week treatment period. Participants in all groups will also receive treatment as usual (TAU) across the study duration. Following the end of 8 week randomised controlled period assessment, participants in all groups will have the option of receiving an additional open label 12 weeks of at-home, remotely supervised tDCS. A follow up assessment will be conducted at 20 weeks post treatment. A flowchart of the study is shown in Fig. 1.

### Eligibility criteria

Male and females will be eligible for enrolment if they meet the following criteria: (1) are aged 16 years or over, (2) have a current Diagnostic and Statistical Manual of Mental Disorders (5th edition DSM-5) diagnosis of anorexia nervosa, (3) are willing and able to participate and comply with study requirements, and (4) worked or studied in a context requiring some proficiency in spoken English (to ensure validity of neuropsychological testing). To ensure patient safety during the study, all participants must be under ongoing care by his/her treating psychiatrist. Potential participants will be excluded in the case that one or more of the following criteria are present: (1) inability to provide informed consent, (2) contraindications to tDCS/rTMS (i.e., metal or devices in head which could interfere with stimulation effects or cause heating, and medical conditions which could increase risk of adverse side effects, e.g., seizures, tinnitus; [44], (3) failed to respond to an adequate course or rTMS (4 weeks) within the current illness course, (4) had ECT in the last 3 months, (5) Montreal Cognitive Assessment (MoCA, Nasreddine, et al. [65]) score of < 26, (6) risk of significant self-harm or suicide as assessed by the study psychiatrist(s), (7) currently enrolled in another interventional clinical trial or using an investigational device/product.

### Recruitment and setting

Participants will be recruited from inpatients in the Eating Disorder Programme for the treatment of anorexia nervosa at Ramsay Northside Clinic, St Leonards, Sydney. The study will recruit 70 participants over a two-year period.

### Interventions

#### Repetitive transcranial magnetic stimulation (rTMS)

rTMS will be administered using a MagPro TMS device. rTMS involves the application of transient magnetic pulses which induce small currents in the underlying cortex via the principal of electromagnetic induction. Both sham and active rTMS will be commenced while participants are inpatients and will continue over the 8 weeks of acute treatment. rTMS will be twice per weekday (separated by ≥ 2 h) [53] over the first 4 weeks (Monday-Friday) and then twice daily per day (separated by ≥ 2 h), given on 2 days each week for the following 4 weeks. Treatment every weekday for 4 weeks is consistent with a typical therapeutic course for intermittent theta-burst stimulation (iTBS) [9]. A taper period over the following 4 weeks is consistent with clinical recommendations for rTMS [59] and has been designed to coincide with participants attending the hospital for day patient programs, 2 days per week, or continuing in outpatient care following discharge from the inpatient program. Active rTMS will be administered using a patterned frequency stimulus called intermittent theta-burst stimulation (iTBS) [40]. This form of rTMS was chosen because a recent large multicentre trial showed 3 min of iTBS attained the same therapeutic effect as 30 min of standard rTMS, leading to FDA approval for depression [9]. Each rTMS treatment session will comprise an extended iTBS session, i.e., 6.6 min, delivered at 100% resting motor threshold (RMT). Extended iTBS has previously been found safe and effective in patients with depression [15]. It will be targeted to the left DLPFC (based on F3 using the 10-20 International EEG system), consistent with the prior RCT of rTMS for AN [20, 21]. For sham rTMS, a sham coil will be placed on the head with no active stimulation administered.

#### Transcranial direct current stimulation (tDCS)

tDCS will be self-administered using the 1 × 1 tDCS mini-CT Stimulator (Soterix, USA: ARTG: 284637) with two saline-soaked sponge electrodes held in place on the scalp using the Soterix Ole-2 headband [72]. The device is intended to treat different neurological and psychiatric disorders. tDCS involves the passing of weak electrical current through the brain via electrodes placed upon the scalp. The current modulates the resting membrane potential of stimulated neurons which causes changes in neuronal excitability. The anode will be placed over the left F3 (10-20 System) and the cathode over F4 (electrode sizes 5 × 5 cm, 25 cm^2^) using the BEAM F3 method [61]. This montage was chosen to target the left DLPFC, consistent with prior studies of tDCS in AN [7, 45, 71]. Sham and active tDCS will be given 84 sessions over 8 weeks of TAU: twice daily (separated by ≥ 2 h) over the first 4 weeks, and daily over the second 4 weeks of the 8 week treatment period. Twice daily tDCS was found safe and effective in previous pilot trial in AN [71]. Active tDCS will be given continuously for 30 min at 2 mA. Sham tDCS will involve an initial ramping up to 1.0 mA and then a ramp down to 0 mA, with a similar ramp up and down at the end of the treatment. Participants receiving active or sham tDCS will self-administer treatment after receiving training, practice and credentialling consistent with our home-based tDCS protocol [3]. The research team will routinely monitor participant’s adherence to the intervention protocol and side effects from each treatment recorded on the Soterix Medical ElectraRx platform. The Soterix Medical ElectraRx solution is a software portal connecting stimulation administrators/clinicians and individuals with the data collected from Soterix Medical neuromodulation technologies including tDCS. At all times, the research team will have real-time access to the information collected by the individual’s device. They will also have access to the individual’s historical data, side effects, stimulation compliance, and warning signs.

### Optional open label active tDCS treatment

Following the end of 8 week randomised controlled period assessment, participants in all groups will have the option of receiving an additional 12 weeks of daily (1 session per day), at-home, remotely supervised active tDCS, until end week 20.

### Screening and enrolment

A study psychiatrist will discuss participation in detail before obtaining written informed consent. Potential participants will be assessed and screened for eligibility before enrolment to the study. Participants’ treating psychiatrist will provide their clinical characteristics including a Diagnostic and Statistical Manual of Mental Disorders, (5th edition, DSM-5) diagnosis, duration of illness and information about medication. Participants will complete the Montreal Cognitive Assessment (MoCa; Nasreddine, et al. [65]) and TMS Adult Safety Screen [44].

### Allocation

Once eligibility criteria are met and informed consent obtained, participants will be randomly allocated to one of four treatment arms: active rTMS or sham rTMS (2:1 ratio) or active tDCS or sham tDCS (2:1 ratio). The R package randomizeR will be used to generate the sequences, based on Efron’s biased-coin design. Generation of the sequences will be carried out by the study statistician. Central randomisation will be implemented using the Research Electronic Data Capture (REDCap) Randomisation Module and the study coordinator will be responsible for assigning each participant to a treatment group prior to commencing research treatment. The randomisation sequence as generated will be imported into REDCap and will consist of a code (e.g. ‘A’, ‘B’, ‘C’ or ‘D’ indicating a treatment arm).

### Outcome measures

Outcome measures and administration timepoints are presented in Table 1.

**Table 1:** Outcome measures of the RCT

Measure	Mode	Baseline	Weeks 1–3	Week 4	Week 8	Weeks 10, 12, 14, 16, 18	Week 20
Core an symptoms							
Eating Disorder Examination Questionnaire, EDE-Q, *total score, subscales scores* [30]	Electronic	✓		✓	✓	✓	✓
**Weight—**BMI, *kg/m*^*2*^		✓		✓	✓		✓
Mood							
Montgomery Asberg Depression Rating Score, MADRS, *total score* [64]	Electronic	✓	✓	✓	✓		✓
Neurocognition							
**Perseveration** Wisconsin Card Sorting Test, WSCT, *numbers of errors* [33]	Computer	✓			✓		✓
**Response inhibition** STROOP Colour Word Test, *response time in milliseconds* [70]	Computer	✓			✓		✓
**Field dependence vs independence** Group Embedded Figures Test, GEFT, *completion time in seconds, total number of correct forms* [75]	Paper	✓			✓		✓
**Attention and cognitive flexibility** Trail Making Test parts A & B, TMT, *completion time in seconds* [66]	Paper	✓			✓		✓
Psychological symptoms							
Depression Anxiety and Stress Scale, DASS-21, *subscales total scores* [54]	Electronic	✓	✓	✓	+ Weeks 5–7✓	✓	✓
Quality of life							
The Assessment of Quality of Life Instrument, AQoL-4D, *total score* [38]	Electronic	✓			✓		✓
Interpersonal relations							
Circumplex Scales of Interpersonal Efficacy, CSIE-32, *subscales total scores* [52]	Electronic	✓			✓		✓
**Experience of treatment** OPTIONAL Semi-structured interview	Zoom				✓		✓
**Assessment of blinding**	Electronic				✓		
Economic outcomes							
Administration cost	Hospital data		✓	✓	✓	✓	✓
Medications			✓		✓		✓
Psychology sessions							✓
Length of stay				✓	✓		
Readmissions							✓

#### Primary outcomes

The efficacy and acceptability of rTMS and tDCS treatments will be this trial’s primary outcomes. The efficacy will be measured by a mean improvement of the Eating Disorder Examination Questionnaire (EDE-Q; Fairburn [30]) from baseline to end of treatment at 8 weeks. The EDE-Q is widely considered the gold standard tool for assessment of eating disorder (ED) psychopathology [1]. It is a self-report measure and includes four sub-scales related to the cognitive features of eating disorders: Restraint, Eating Concern, Shape Concern and Weight Concern. This measure for assessing change in the severity of eating disorder psychopathology was chosen due to its sensitivity in recognising behavioural and cognitive change in the recovery for AN [18]. Number of completed sessions for active tDCS and active rTMS in the acute 8-week RCT period will indicate acceptability of both treatments.

#### Secondary outcomes

A range of secondary outcome measures will be administered to further determine rTMS and tDCS treatment effects on: (1) weight, (2) mood, (3) neurocognition (attention and cognitive flexibility, field dependence vs independence, response inhibition, perseveration), (4) psychological symptoms, (5) quality of life, (6) interpersonal relations, (7) experience of treatment (optional semi-structured interviews), (8) economic outcomes (health sector costs including rTMS and tDCS administration, medications, psychology sessions, length of stay, and number of hospital readmissions). See Table 1 for measures.

### Optional interview

Between weeks six and eight, semi-structured qualitative interviews will be offered to all participants to share their views on the experience, and perceived barriers and facilitators for treatment change with rTMS and tDCS. Participants who agree to participate will be interviewed on a one-to-one basis via videoconferencing, following a semi-structured guide (adapted version of topic guide developed by Dalton et al. [19]). All interviews will be audio recorded and will last 10–20 min. Participants will be blinded to the treatment allocation for the first interview (between weeks 6–8 of treatment) and they will be unblinded for the second interview (at week 20 follow up) when they will have an opportunity to share their experience of at home self-administered tDCS.

### Blinding

In this double-blind trial, participants, their treating psychiatrist, ward staff, and a study staff member (who will conduct blinded assessments of mood secondary outcome measures) will be blinded after assignment to an initial intervention until the database is locked and the primary analysis completed. The integrity of blinding will be evaluated at the end of randomised treatment assessment (at 8 weeks follow up) by asking participants and the mood rater conducting the secondary mood outcome rating to indicate which treatment group they thought the participant was allocated to (active tDCS, sham tDCS, active rTMS or sham rTMS) and their confidence (rated 0% = not confident to 100% confident).

### Adverse event monitoring

Participants in all treatment arms will complete a side effects questionnaire after every treatment session, the rTMS group via RedCap survey and tDCS group directly via ElectraRx. Trained staff who administer all rTMS sessions will also record adherence to the intervention protocol. Participants’ adherence to tDCS will be monitored by the tDCS device which records of the number of treatments administered. The research team will routinely monitor participant’s adherence to the intervention protocol and side effects from each treatment.

### Ethical considerations

Reasonable precautions against harms and steps to mitigate risks will be taken. To minimise the potential risk for adverse effects, participants will be screened for potential contraindications for both tDCS and rTMS treatment prior to providing informed consent. Safety and adverse effects will be closely monitored by study staff over the course of the trial and through ongoing care by the participants’ treating psychiatrist, who will continue to oversee clinical progress and the welfare of his/her patient over the course of the study. Every 6 months reports of any serious adverse events will be monitored by an independent medical monitor.

To ensure confidentiality, all data collected will be de-identified. Where participant consent is provided for data to be re-used and shared with other researchers for future research projects, data will only be provided in a de-identified format and with separate ethics approval. No identifiable information will be reported in any format, thus protecting the confidentiality of participants and other information collected during the study.

### Analysis

#### Populations

The analysis will be completed for the Modified Intention to Treat (mITT) and the Per Protocol (PP) populations. The mITT population will include all participants randomised and who had at least 1 randomised treatment and 1 post baseline rating. The PP population is a subset of the mITT population and will include all participants randomised who fulfil the following criteria: (1) received at least 80% of randomised acute active or sham tDCS or active or sham rTMS treatments over the 8 week treatment period and (2) have an absence of significant protocol deviations (received correct treatment assignment in randomised phase and rTMS dose is correct in at least 80% of randomised treatments).

#### Primary outcomes

The efficacy analysis will be completed for both the mITT and PP population. Descriptive statistics and graphical methods will be used to show variability in the data. The size of the treatment effect for EDE-Q Global scores will be the change in scores from pre-treatment to post 8 weeks treatment for both active treatment arms. The acceptability analysis will be completed in the mITT population. Descriptive statistics will report the number of completed active tDCS and active rTMS treatments in the 8 week randomised controlled period.

#### Secondary outcomes

Analyses of secondary outcomes will be competed in the mITT population. The size of the treatment effect will be the difference in outcome data (BMI, questionnaires, neurocognitive outcomes) between the active and sham treatment arms for both tDCS and rTMS, respectively, at 4 weeks, 8 weeks, and 20 weeks post treatment. These effect sizes will control for pre-treatment scores. Descriptive statistics will be used to show remission rates for all treatment arms at post 8 weeks and 20 weeks post treatment. Generalised linear mixed models will be used to compare the treatment arms for mood and psychological symptom outcomes. Exploratory analyses will investigate associations between changes in mood and neurocognition with active tDCS and rTMS, and improvement in core eating disorder symptoms.

Generalised linear models will be used to compare cost and quality of life/utility values (from AQoL-4D) between treatments arms over the 8 week randomised treatment. Cost effectiveness analysis will compare the health outcomes and economic outcomes (costs of duration of inpatient stay and readmissions during the follow up period) associated with both treatment modalities.

#### Qualitative data

Since thematic saturation may be achieved in 12 interviews [36], our aim is to collect qualitative data for 10–20 participants from each treatment group. Audio-recorded interviews will be transcribed verbatim in accordance with standardised guidelines [60] and will be analysed using inductive approach qualitative methods with use of the framework of thematic analysis [11]. This will include coding and generation of themes from participants accounts of their experience of rTMS and tDCS treatment.

## Discussion

AN has limited treatment outcomes, and the highest treatment costs of any psychiatric disorder [35]. This study is designed to investigate and compare relative acceptability and efficacy of two non-invasive forms of brain stimulation: tDCS and rTMS, which haven shown a potential to reducing core ED symptoms in AN [29].

Currently, one randomised sham controlled clinical trial each investigating tDCS [7] and rTMS [20, 21] have indicated limited efficacy for core AN symptoms, with the tDCS study observing reduction in need for excessive control over food intake and improvement in body image and the rTMS study observing improvements in psychological symptoms (anxiety and depression) and in quality of life. In these studies, the overall number of treatment sessions has been limited (i.e., 10 tDCS, 20 rTMS) which contrasts with standard therapeutic treatment protocols for other indications, for example depression [63]. This study will directly compare the therapeutic efficacy and acceptability of both treatment modalities with an increased number of treatments (tDCS 84 and rTMS 56 sessions) over a longer sham-controlled treatment period (8 weeks).

When designing the study, the potential burden on participants was considered due to the inclusion of an increased number of treatments compared to prior studies and the longer sham-controlled period. To minimise this burden, it was first decided to schedule the treatments around the participants’ inpatient and outpatient program at the hospital to minimise travel burden. Second, we implemented an overall 2:1 active to sham randomisation so that participants are twice as likely to receive active treatment in the sham controlled RCT phase of the study. Another strategy was to provide all participants who complete the first 8 weeks RCT the option of receiving 12 weeks of open label active home based tDCS treatment, i.e. the study provided the option of extended active tDCS treatment to all participants.

AN patients in previous studies were either in-patients receiving pharmacotherapy and psychotherapy [7] or out-patients with or without ED treatment [20, 21]. In this study, all participants will start as inpatients who will be receiving intensive concomitant treatment, i.e., comprehensive in-patient program with individual medication and/or psychotherapy and group cognitive behavioural therapy (CBT). This study will therefore explore, for the first time, the potential for additional therapeutic benefits of brain stimulation combined with existing eating disorders interventions.

AN has one of the highest treatment costs of any psychiatric disorder, largely due to the high cost of recurrent hospitalisation for nutritional rehabilitation [35]. Our study will explore the potential for these treatment modalities to reduce the length of hospital stays and emergency readmissions. Health economic data for both treatment modalities will additionally have utility from a service perspective, given the disparity in resource requirements between the two treatments (TMS, tDCS) in terms of costs for patients and access to treatment for people living in remote and rural areas (i.e., for at-home tDCS).

AN has been associated with impaired quality of life and lower preference-based health state utility values [2, 42, 49]. Utility values are used to calculate quality adjusted life years (QALYs) and used as an outcome measure by health technology assessment agencies such as the Pharmaceutical Benefits Advisory Committee in Australia and The National Institute for Health and Care Excellence in the UK. Our analysis will investigate the potential for these treatments to improve the quality of life and health state utility values for people with AN. This data will be useful for input into future trials and model based economic evaluations of these treatments.

Future larger scale multicentre clinical trials should be designed to determine the optimal number and duration of non-invasive brain stimulation treatments for AN. The possible use and comparison of personalized protocols can provide valuable insight as it has been shown that TMS, derived from various neuroimaging techniques, tend to be more effective than standard TMS in the treatment of depression [62]. The rTMS/tDCS treatment target guided by MRI [13] could also potentially increase treatment efficacy in AN. Future trials should include even longer-term follow-up to determine if positive clinical outcomes are maintained following neuromodulation treatment or if these effects diminish with time.

In summary, research into non-invasive brain stimulation as treatments for AN has potential to improve clinical outcomes for patients by comparing the relative efficacy and acceptability of both treatment modalities in the inpatient and at-home setting (i.e., for at-home tDCS) results from this study will provide important information for informing future larger clinical trials of these treatments for AN.

### Trial status

Clinical Trial Protocol version 8 dated 11 July 2023. Recruitment commenced in August 2023 and will end in May 2025.

## Data Availability

The TRENA trial coordinating team will oversee data management with input from trial statisticians. UNSW will maintain ownership of all study data collated for analysis. Data access will be managed by the TRENA trial coordinating team and governed by the Trial Steering Group.

## Abbreviations

AN: Anorexia Nervosa
AQoL-4D: The Assessment of Quality of Life Instrument
CSIE-32: Circumplex Scales of Interpersonal Efficacy
DASS-21: Depression Anxiety and Stress Scale
DLPFC: Dorsolateral Prefrontal Cortex
DMPFC: Dorsomedial Prefrontal Cortex
DSM-5: Diagnostic and Statistical Manual of Mental Disorders (5th edition)
ED: Eating Disorder
EDE-Q: Eating Disorder Examination Questionnaire
EEG: Electroencephalography
FDA: Food and Drug Administration
GEFT: Group Embedded Figures Test
iTBS: Intermittent Theta Burst Stimulation
MADRS: Montgomery Asberg Depression Rating Score
mITT: Modified Intention to Treat
MoCA: Montreal Cognitive Assessment
PP: Per Protocol
RCT: Randomised Controlled Trial
REDCap: Research Electronic Data Capture
rTMS: Repetitive Transcranial Magnetic Stimulation
TAU: Treatment As Usual
tDCS: Transcranial Direct Current Stimulation
TMT: Trail Making Test
WSCT: Wisconsin Card Sorting Test

## Declarations

### Confidentiality

To ensure confidentiality, all data collected will be de-identified. Research data will only be accessible to staff involved in the study as needed. Participants’ study information will not be released outside of the study without the written permission of the participant, except as required by the HREC, sponsor, or regulatory authorities. Where participant consent is provided for data to be re-used and shared with other researchers for future research projects, data will only be provided in a de-identified format and with separate ethics approval.

### Ancillary and Post-Trial Care

Reasonable precautions against harms and steps to mitigate risks will be taken. In the trial protocol, safety will be closely monitored through ongoing care by the patients’ treating psychiatrist who will continue to oversee clinical progress and the welfare of his/her patient. Either the participant or his/her treating/admitting psychiatrist can withdraw the participant from the study at any time if there are concerns about clinical progress or welfare. Post-trial care will not be provided by study staff or investigators.

### Dissemination Policy

Results of the research will be disseminated via publication of papers in academic journals, reports, and presentations at academic meetings and conferences. Only aggregated group (or subgroup) results will be analysed and reported as such. No identifiable information will be reported in any format, thus protecting the confidentiality of participants and other information collected during the study.
